# Epidemiology of GII.4 and GII.2 norovirus outbreaks in closed and semi-closed institutions in 2017 and 2018

**DOI:** 10.1038/s41598-023-28448-9

**Published:** 2023-01-30

**Authors:** Thais Cornejo-Sánchez, Núria Soldevila, Lorena Coronas, Miquel Alsedà, Pere Godoy, Efrén Razquín, Sara Sabaté, Susana Guix, Virginia Rodríguez Garrido, Rosa Bartolomé, Angela Domínguez, the Working Group for the Study of Outbreaks of Acute Gastroenteritis in Catalonia, Josep Álvarez, Anna Isabel Belver, Neus Camps, Sofia Minguell, Monica Carol, Conchita Izquierdo, Ignacio Parrón, Cristina Pérez, Ariadna Rovira, Maria Sabaté, Maria Rosa Sala, Rosa Maria Vileu, Irene Barrabeig, Mireia Jané, Ana Martínez, Núria Torner, Javier de Benito, Antonio Moreno-Martínez, Cristina Rius, Anna de Andres, Esteve Camprubí, Montse Cunillé, Maria Lluïsa Forns, Mercé de Simón

**Affiliations:** 1 Laboratori de Microbiologia, Hospital Universitari Vall d’Hebrón, Barcelona, Spain; 2 CIBER Epidemiología y Salut Pública (CIBERSP), Madrid, Spain; 3 Departament de Medicina, Universitat de Barcelona, Barcelona, Spain; 4 Agència de Salut Pública de Catalunya, Barcelona, Spain; 5 Institut de Recerca Biomédica de Lleida, IRB Lleida, Lleida, Spain; 6 Agència de Salut Pública de Barcelona (ASPB), Barcelona, Spain; 7 Departament de Genètica Microbiologia i Estadística, Grup de Virus Entèrics, Universitat de Barcelona, Barcelona, Spain; 8 Nutrition and Food Safety Research Institute (INSA·UB), Universitat de Barcelona, 08921 Santa Coloma de Gramenet, Spain

**Keywords:** Gastroenteritis, Epidemiology

## Abstract

Norovirus infections are a leading cause of acute gastroenteritis outbreaks worldwide, with genotypes GII.2 and GII.4 being the most prevalent. The aim of this study was to compare the characteristics of GII.2 and GII.4 norovirus outbreaks reported in Catalonia in closed or semi-closed institutions in 2017 and 2018. The epidemiological and clinical characteristics of GII.2 and GII.4 outbreaks were compared using the chi-square test or Fisher's exact test for categorical variables and the Mann–Whitney U test for continuous variables. Odds ratios and their 95% confidence intervals were estimated. 61 outbreaks were reported: GII.4 was the causative agent in 12 outbreaks (30%) and GII.2 in 9 outbreaks (22.5%). GII.2 outbreaks were detected more frequently in schools or summer camps (66.7%) and GII.4 outbreaks in nursing homes (91.7%) (*p* = 0.01). Ninety-three people were affected in GII.2 outbreaks and 94 in GII.4 outbreaks. The median age was 15 years (range: 1–95 years) in GII.2 outbreaks and 86 years (range: 0–100 years) in GII.4 outbreaks (*p* < 0.001). Nausea, abdominal pain, and headache were observed more frequently in persons affected by GII.2 outbreaks (*p* < 0.05). Symptomatic cases presented a higher viral load suggestive of greater transmission capacity, although asymptomatic patients presented relevant loads indicative of transmission capacity.

## Introduction

Following the introduction of European Community Regulation (EC) No 2160/2003 of the European Parliament and of the Council of 17 November 2003 on the control of *Salmonella* and other zoonotic agents^1^, the number of outbreaks of acute gastroenteritis (AGE) caused by *Salmonella* spp. has decreased considerably in Spain. However, norovirus as a cause of AGE has increased in recent years, which may be attributed to high sensitivity detection techniques and the improved ability of public health agencies to detect and study AGE outbreaks thanks to technological advances^2^. It is difficult to determine the exact number of norovirus gastroenteritis cases, since many affected people do not visit their doctor as they only have mild symptoms, but it is estimated that currently there are about 685 million cases per year worldwide^3^.

Norovirus is a virus of the *Caliciviridae* family and is currently classified into 10 genogroups (GI to GX) of which norovirus GI, GII, GIV, GVIII and GIX are known to affect humans^4^. These genogroups are, in turn, classified into genotypes defined by a region of the genome encoding for the VP1 capsid protein. Nine genotypes have been described for norovirus GI, 27 for norovirus GII and 2 for norovirus GIV^5^. Studies from different geographical regions agree that norovirus GII.4 is the most persistent genotype over time and the most prevalent in some regions^6–9^. A study by Motoya et al*.* of the epidemiology of norovirus in Japan from 2012 to 2018 found norovirus GII.4 was the most prevalent genotype until 2015, when the same number of norovirus samples of GII.4 and GII.17 were detected. In 2016 there was a considerable increase in norovirus GII.2, which became the most prevalent. However, norovirus GII.4 increased again in 2018^10^.

Norovirus transmission routes include food, contaminated water, person-to-person contact^11,12^ and fomites. Norovirus can remain active in the environment for a long period and is relatively resistant to some disinfection products and methods^12^. The infectious dose of norovirus has been described as 10–100 viral particles^12^. These features and the ability to mutate make it a highly transmissible virus, especially in closed or semi-closed institutions where shared toilets and other areas may facilitate transmission^13^. According to the US Centers for Disease Control and Prevention (CDC), from 2009 to 2013, 62.5% of outbreaks caused by norovirus occurred in long-stay health centers, similar to the results reported in the European Union^14,15^.

The objective of this study was to determine the involvement of norovirus in producing AGE outbreaks in closed or semi-closed institutions in Catalonia in 2017 and 2018 and analyze the clinical and epidemiological characteristics of the most prevalent genotypes (GII.4 and GII.2).

## Material and methods

Outbreaks of acute AGE reported to the Public Health Agency of the Generalitat of Catalunya that tested positive for norovirus and occurred in closed or semi-closed institutions were selected. A closed or semi-closed institution was defined as one in which people generally stay overnight for a certain period or in which people are confined for a certain time. The institutions considered were nursing homes, nurseries, preschool and school centers, children's clubs, summer camps and hotels.

An outbreak was defined as ≥ 2 patients with symptoms of AGE who coincided in space and time. Once outbreaks were reported to the Epidemiological Surveillance units of the Public Health Agency of Catalonia, public health technicians obtained data on the study variables through an epidemiological survey of the people involved, and stool samples were collected for microbiological study. At the end of the outbreak, public health technicians prepared a technical report on each outbreak, collecting information on the most relevant variables (genotype, scope, mode of transmission and season). April to September were considered the warm or temperate months and October to March as the winter months. The month of notification of each outbreak was categorized as "warm season" or "winter season".

Fecal samples of outbreaks in Barcelona city were sent to the Public Health Agency of Barcelona laboratory (ASPB) and outbreaks in the rest of Catalonia were analyzed in the laboratory for the study of community outbreaks of food poisoning of the Vall d'Hebron University Hospital.

Enteric viruses were detected using real-time PCR with the Allplex™ GI-Virus Assay (Seegene, Inc.), which simultaneously detects norovirus GI, norovirus GII, adenovirus, rotavirus, sapovirus and astrovirus in Vall d’Hebron University Hospital and viral RNA was extracted from a 10% stool suspension using the NucliSENS^®^ easyMAG^®^ system (BioMérieux, Marcy-L’Etoile, France), and the presence of HuNoV was assessed by RTqPCR according to ISO 15216-2:2019^16^ in ASPB laboratory. Samples of positive for norovirus GI and/or norovirus GII were genotyped by nested RT-PCR and sequencing the amplicon, following the protocol described by van Beek et al.^17^ Amplification was performed by RT-PCR using the One-Step RT-PCR kit (Qiagen, Hilden, Germany). Sequences were obtained using the ABI 3730 platform (Applied Biosystems, Foster City, California, USA) and were assembled with the SeqMan 4.05 program (Dnastar, Madison, WI, USA). Once the sequences were obtained, the genotype was obtained using the Norovirus Typing Tool Version 2.0 (https://www.rivm.nl/mpf/typingtool/norovirus/). The real-time PCR cycle threshold (Ct) was used to estimate the semi-quantitative determination of the viral load in the samples processed.

The statistical analysis was carried out using the chi-square test or Fisher's exact test for categorical variables, Fisher’s exact test was used when the cell had an expected frequency less than 5, and the Mann–Whitney U test for continuous variables. Crude and adjusted odds ratios (OR) and their 95% confidence intervals (CI) were estimated using logistic regression. Adjusted OR (aOR) were adjusted for age and sex. A *p* value < 0.05 was considered statistically significant. The analysis was made using the SPSS v.25 statistical package.

### Ethics declarations and informed consent statement

The study was conducted according to the guidelines of the Declaration of Helsinki, regulations of the Public Health Agency of Catalonia and ethical protocols established. The study was approved by the University of Barcelona Bioethics Commission (ethics approval number IRB00003099) on April 12, 2016.

The authors declare that the Bioethics Committee of University of Barcelona approved the waiver for informed consent. All data used in the analysis were collected during routine public health surveillance activities as part of the legislated mandate of the Health Department of Catalonia, which is officially authorized to receive, treat and temporarily store personal data in the case of infectious disease. All data were fully anonymized. All study activities formed part of the public health surveillance tasks. The law regulates these activities and informed consent should not be necessary.

## Results

From 1 January 2017 to 31 December 2018, 61 outbreaks of AGE positive for norovirus were reported in closed or semi-closed institutions: of these, 40 (65.5%) were positive for GII, 14 (23%) for GI and 7 (11.5%) for both genogroups. Of the 40 positive outbreaks for GII, GII.4 was responsible in 12 outbreaks (30%), GII.2 in 9 outbreaks (22.5%), GII.17 in 7 outbreaks (17.5%), GII.6 in 5 outbreaks (12.5%), and GII.1 in 1 outbreak (2.5%). In 6 outbreaks (15%) the genotype could not be determined, either because we did not obtain PCR products or the quality and/or the size of the sequences obtained from these outbreak’s samples were not enough to determine the genotype (Table 1). The genotypes responsible for the 14 most frequent GI-positive outbreaks were GI.4 in 5 outbreaks (35.7%) and GI.1 in 3 outbreaks (21.4%) (Table 1).

**Table 1:** Distribution of genotypes of outbreaks of acute norovirus gastroenteritis.

Genotypes	N (%)
Genogroup I	14
GI.1	3 (21.4%)
GI.3	2 (14.3%)
GI.4	5 (35.7%)
GI.5	1 (7.1%)
GI.6	2 (14.3%)
GI.2 and GI.3	1 (7.1%)
Genogroup II	40
GII.1	1 (2.5%)
GII.2	9 (22.5%)
GII.4	12 (30%)
GII.6	5 (12.5%)
GII.17	7 (17.5%)
Sequences could not be obtained	6 (15.0%)

Thirty-nine of the 61 outbreaks (63.9%) occurred in the winter months. Table 2 shows the distribution of outbreaks caused by norovirus in the warm and winter season according to the location of the outbreak.

**Table 2:** Distribution of the location of outbreaks according to the season.

Season	Areas	N (%)
Warm	Summer camp	7 (31.8%)
	Nursing home	5 (22.7%)
	Hotel	3 (13.6%)
	School	3 (13.6%)
	Social health center	2 (9.1%)
	Hospital	1 (4.5%)
	Nursery	1 (4.5%)
	Total	22 (100%)
Winter	Nursing home	25 (64.1%)
	School	7 (17.9%)
	Summer camp	3 (7.7%)
	Nursery	2 (5.1%)
	Hotel	1 (2.6%)
	Social health center	1 (2.6%)
	Total	39 (100%)

The characteristics of GII.2 and GII.4 outbreaks are shown in Table 3. Norovirus GII.2 outbreaks were more frequently detected in schools or summer camps (66.7%) and norovirus GII.4 outbreaks in nursing homes (91.7%) (*p* = 0.01). The mode of transmission was person-to-person in 55.6% of norovirus GII.2 outbreaks and 100% in norovirus GII.4 outbreaks (*p* = 0.02): 91.7% of norovirus GII.4 outbreaks and 44.4% of norovirus GII.2 outbreaks occurred in the winter season (*p* = 0.05).

**Table 3:** Characteristics of outbreaks caused by norovirus GII.2 and GII.4 according to location, mode of transmission and season.

Features	GII.2	GII.4	*p* value
Total	9	12	
Location			0.01
School/nursery	2 (22.2%)	1 (8.3%)	
Summer camp	4 (44.4%)	0 (0%)	
Nursing home	2 (22.2%)	11 (91.7%)	
Hotel	1 (11.1%)	0 (0%)	
Mode of transmission			
Person-to-person	5 (55.6%)	12 (100%)	0.02
Foodborne	4 (44.4%)	0 (0%)	
Season			0.05
Winter	4 (44.4%)	11 (91.7%)	
Warm	5 (55.6%)	1 (8.3%)	

A total of 809 surveys of symptomatic patients were obtained in the 61 outbreaks: 537 (66.8%) were female and 267 (33.2%) male. The median age was 41 years (range: 6 months–105 years). Information on the duration of symptoms was available in 497 surveys, the median duration of symptoms was 1 day (range: 0–30 days); 21.7% (108/497) of those affected had symptoms of < 24 h of evolution, 30.6% (152/497) of one day of evolution, 21.7% (108/497) of 2 days of evolution, 14.1% (70/497) of 3 days of evolution and 11.9% (59/497) had a duration of ≥ 4 days. The most frequent symptoms were vomiting (80.1%), diarrhea (74%), abdominal pain (62.4%), nausea (55.7%) and fever (27.6%). Information on required oral fluid replacement, intravenous fluid replacement and hospital admission was available in 397, 361 and 752 surveys respectively, 23.4% (93/397) required oral fluid replacement, 4.7% (17/361) intravenous fluid replacement and 2.5% (19/752) hospital admission. No cases were admitted to ICU nor died.

Ninety-three people were affected in GII.2 outbreaks and 94 in GII.4 outbreaks. GII.4 outbreaks had a higher frequency of women than GII.2 outbreaks (79.8% vs. 59.8), although the differences were not statistically significant in the multivariate analysis (Table 4). The median age was 15 years (range: 1–95 years) in those affected by GII.2 outbreaks and 86 years (range: 0–100 years) in those affected by GII.4 outbreaks (*p* < 0.001). Fever, nausea, abdominal pain, headaches, chills, and myalgia were observed more frequently in people affected by GII.2 outbreaks than in those affected by GII.4 outbreaks (*p* < 0.05). The duration of symptoms was longer in people affected by GII.4 than in those affected by GII.2 (median of 1 day vs. median of 2 days, *p* < 0.001). Those affected by GII.2 outbreaks had more hospitalizations than those affected by GII.4 outbreaks (10.8% vs. 2.4%), although the differences were not statistically significant in the adjusted analysis (Table 4). People hospitalized due to GII.2 outbreaks were aged 15–16 years who were basketball players from another autonomous community and were admitted to a private clinic; of the two people hospitalized due to GII.4 outbreaks one was aged 93 years while the age was not known in the second, although he was known to be a nursing home worker.

**Table 4:** Characteristics persons affected by GII.2 and GII.4 outbreaks.

Characteristics	GII.2	GII.4	Crude OR (95% CI)	*p* value	Adjusted OR (95% CI)	*p* value
Total	93	94				
Sex						
Male	37/92 (40.2%)	19/94 (20.2%)	2.65 (1.38–5.10)	0.003	0.87 (0.31–2.41)	0.79
Female	55/92 (59.8%)	75/94 (79.8%)	Ref		Ref	
Age-median (range)	15 (1–95)	86 (0–100)				
0–4 years	8/93 (8.6%)	6/85 (7.1%)	6.13 (1.85–20.30)	0.003	6.30 (1.87–21.20)	0.003
5–15 years	41/93 (44.1%)	1/85 (1.2%)	188.60 (24.02–1480.94)	< 0.001	191.52 (23.80–1540.87)	< 0.001
16–39 years	18/93 (19.4%)	2/85 (2.4%)	41.40 (8.66–197.79)	< 0.001	44.68 (8.46–236.01)	< 0.001
40–64 years	11/93 (11.8%)	7/85 (8.2%)	7.22 (2.13–25.24)	< 0.001	7.22 (2.40–21.71)	< 0.001
> 64 years	15/93 (16.1%)	69/85 (81.2%)	Ref		Ref	
Viral load (CT) median (range)	23.82 (17.56–43.48)	24.31 (16.08–36.69)	0.99 (0.93–1.06)	0.93	0.97 (0.88–1.07)	0.51
Duration of symptoms-median (range)	1 (0–4)	2 (0–7)	0.55 (0.39–0.76)	0.01	0.41 (0.27–0.64)	< 0.001
Symptoms^a^						
Fever	39/83 (47.0%)	6/54 (11.1%)	7.09 (2.74–18.37)	< 0.001	2.78 (0.87–8.91)	0.08
Diarrhea	70/89 (78.7%)	54/62 (87.1%)	0.55 (0.22–1.34)	0.18	0.85 (0.28–2.55)	0.77
Nausea	66/87 (75.9%)	13/53 (24.5%)	9.67 (4.36–21.42)	< 0.001	8.93 (3.36–23.72)	< 0.001
Vomiting	77/92 (83.7%)	46/65 (70.8%)	2.12 (0.98–4.58)	0.05	2.32 (0.99–5.13)	0.05
Abdominal pain	75/88 (85.2%)	19/58 (32.8%)	11.84 (5.30–26.48)	< 0.001	8.73 (3.34–22.87)	< 0.001
Headaches	34/80 (42.5%)	2/48 (4.2%)	17.00 (3.86–79.94)	< 0.001	6.64 (1.39–31.75)	0.02
Chills	9/55 (16.4%)	0/41 (0%)	–	0.01	–	
Myalgia	7/56 (12.5%)	0/41 (0%)	–	0.02	–	
Vertigo	2/47 (4.3%)	0/41 (0%)	–	0.50	–	
Lethargy	0/45 (0.0%)	1/41 (2.4%)	–	0.48	–	
Malaise	10/11 (90.9%)	8/8 (100%)	–	1.00	–	
Hospitalization						
Yes	8/74 (10.8%)	2/83 (2.4%)	4.91 (1.01–23.91)	0.04	3.51 (0.29–42.99)	0.32
No	66/74 (89.2%)	81/83 (97.6%)	Ref		Ref	

There were 64 asymptomatic cases, 10 occurred in GI outbreaks, 40 in GII outbreaks and 14 in GI and GII outbreaks. Of the 40 asymptomatic people associated with GII norovirus outbreaks, 28 (70.0%) were female and the median age was 46 years. Of these, 6 were involved in GII.2 outbreaks and 9 in GII.4 outbreaks. Comparison of the characteristics of symptomatic persons affected by GII, GII.2 and GII.4 outbreaks with those of asymptomatic persons (Table 5) showed significant differences in age: in GII outbreaks, the most frequent age groups in affected symptomatic people were the > 64 years (34.6%) and 5–15 years (31.3%) age groups, while asymptomatic patients were more frequent in the 46–64 years (35.0%) and > 64 years age groups (25.0%). In GII outbreaks, the Ct was higher in asymptomatic than in symptomatic persons (median 25.16 vs. 23.74, *p* < 0.01), although the differences were not statistically significant in GII.2 and GII.4 outbreaks, probably due to the small number of patients (Table 5).

**Table 5:** Characteristics of symptomatic and asymptomatic persons affected by norovirus GII, GII.2 and GII.4 outbreaks.

Features	Symptomatic	Asymptomatic	Crude OR (95% CI)	*p* value	Adjusted OR (95% CI)	*p* value
All norovirus GII	463	40				
Sex						
Male	161(35.2%)	12 (30.0%)	1.26 (0.63–2.56)	0.51	1.31 (0.63–2.71)	0.47
Female	297(64.8%)	28 (70.0%)	Ref		Ref	
Age-median (range)	24 (0–105)	46 (0–95)		0.01		
Age groups						
0–4 years	53 (11.8%)	8 (20.0%)	0.43 (0.16–1.14)	0.09	0.41 (0.15–1.11)	0.08
5–15 years	140 (31.3%)	1 (2.5%)	9.03(1.14–71.46)	0.04	9.00(1.12–72.15)	0.04
16–39 years	60(13.4%)	7 (17.5%)	0.55 (0.20–1.52)	0.25	0.56 (0.20–1.57)	0.27
40–64 years	40 (8. 9%)	14(35.0%)	0.18 (0.08–0.45)	< 0.01	0.18 (0.08–0.44)	< 0.01
> 64 years	155 (34.6%)	10 (25.0%)	Ref		Ref	
Viral load-median Ct (range)	23.74 (14.04–43.48)	25.16 (16.67–45.11)	0.91 (0.86–0.96)	0.02	0.92 (0.87–0.98)	0.01
Norovirus GII.2	93	6				
Sex						
Male	37 (40.2%)	1 (16.7%)	3.36 (0.38–29.97)	0.40	2.18 (0.22–21.18)	0.50
Female	55 (59.8%)	5 (83.3%)	Ref		Ref	
Age-median (range)	15 (1–95)	58 (31–85)		0.01		
Age groups						
0–4 years	8 (8.6%)	0 (0%)	–	–	–	
5–15 years	41 (44.1%)	0 (0%)	–	–	–	
16–39 years	18 (19.4%)	1 (16.7%)	2.40 (0.20–19.13)	0.49	1.74 (0.11–26.60)	0.69
40–64 years	11 (11.8%)	3 (50.0%)	0.49 (0.07–3.44)	0.47	0.50 (0.07–3.51)	0.48
> 64 years	15 (16.1%)	2 (33.3%)	Ref		Ref	
Viral load-median Ct (range)	23.82 (17.56–43.48)	27.34 (19.52–36.20)	0.90 (0.78–1.03)	0.11	0.94 (0.81–1.09)	0.94
Norovirus GII.4	94	9				
Sex						
Male	19 (20. 2%)	3 (33.3%)	0.51 (0.12–2.21)	0.37	0.53 (0.13–2.87)	0.65
Female	75 (79.8%)	6 (66.7)	Ref		Ref	
Age-median (range)	86 (0–100)	33 (0–88)		0.01		
Age groups						
0–4 years	6 (7.1%)	4 (44.4%)	0.04 (0.01–0.29)	< 0.01	0.04 (0.01–0.29)	< 0.01
5–15 years	1 (1.2%)	0 (0%)	–	–	–	–
16–39 years	2 (2.4%)	2 (22.2%)	0.03 (0.01–0.32)	< 0.01	0.03 (0.01–0.31)	< 0.01
40–64 years	7 (8.2%)	1 (11.1%)	0.20 (0.02–2.53)	0.22	0.20 (0.02–2.46)	0.21
> 64 years	69 (81.2%)	2 (22.2%)	Ref		Ref	
Viral load-median Ct (range)	24.31 (16.08–36.69)	27.16 (19.74–40.00)	0.90 (0.80–1.04)	0.13	0.84 (0.69–1.02)	0.08

## Discussion

In the 61 AGE outbreaks in closed or semi-closed institutions in Catalonia that tested positive for norovirus in 2017 and 2018, GII was the most frequently detected genogroup.

Studies have shown that a large percentage of infections caused by norovirus occur in the winter months^18–21^, although other studies did not observe this^22^. We found 63.9% of outbreaks occurred in the winter months, but many outbreaks were also detected in the warmer months, which occurred mainly in places where there is more movement of people, such as hotels and summer camps.

Females accounted for 66.8% of affected persons and the duration of the most frequent acute symptoms was one day (30.6%), although symptoms extended to > 4 days in some cases. The most common symptoms were vomiting, diarrhea, and abdominal pain: 4.7% of affected people required intravenous fluid replacement and 2.5% hospital admission.

The genotypes causing the most outbreaks were GII.4, followed by GII.2. Although norovirus GII.4 has remained the majority genotype over time and in several regions^11,23,24^, the sporadic emergence of some genotypes such as GII.17 or, more recently, the recombinant genotype GII.2[P16]^25^ has been observed. We could not determine the permanence of GII.4 in the time period studied but, compared with a Catalonian study in 2010–2012^11^, an increase in outbreaks caused by GII.4 was observed, which could indicate a trend to reemergence. The conclusions presented in the work carried out by Graaf et al. suggest that the high mutation rate of GII.4 could explain the permanence of this genotype^8^. All but one GII.4 outbreaks were detected in the winter months. The 2010–2012 study in Catalonia, which studied outbreaks in all areas, including closed and semi-closed institutions, found that norovirus GII.4 was detected mostly in the winter months (83.3%)^11^. In study carried out in USA during 2012–2015 found that GII.4 noroviruses had significant peaks during winter months^26^. For norovirus GII.2 no seasonal pattern has been observed, but the fewer outbreaks due to this genotype and the fact that most outbreaks occurred in summer camps, which take place in the warm season, may explain why seasonality has not been detected. In Germany during the 2016 winter, the GII.2 was dominant in the reported outbreaks^27^.

Outbreaks caused by GII.4 were detected almost entirely in nursing homes and transmission was person-to-person, while outbreaks due to GII.2 were detected in all types of closed and semi-closed centers, but more frequently in summer camps, and the mode of transmission was both person-to-person and foodborne. Coinciding with some reported results, in our study norovirus GII.4 mostly affected adolescents and adults, while GII.2 affected children to a greater extent^28,29^. This may be explained by the fact that adults have been creating immunity against different genotypes, except against the different gene varieties produced by norovirus GII.4^30^.

The duration of symptoms was longer in infections caused by norovirus GII.4, but the only clinical manifestations which had a differing frequency in the genotypes studied were nausea, abdominal pain, and headache, which were more frequent in cases produced by GII.2. Wang et al*.* in a study carried out in Shanghai schools in 2017 found no differences between these genotypes in abdominal pain^31^. Matthew et al. in a study carried out in hospitalized children in Qatar between June 2016 and June 2018 classified the disease severity produced by the different genotypes and found that these two genotypes produce moderate severity^32^.

The median age in symptomatic patients was higher in people infected with norovirus GII.4 compared with those affected with GII.2. The comparison of these two genotypes shows that, although there were no significant results, there was a trend for people infected with norovirus GII.2 to have a greater requirement for hospitalization despite being much younger and with less morbidity. Our results show that 80% of hospitalizations were in people aged 15–16 years. Other authors^33^ have found that norovirus causes more complications and hospitalizations in children, with a higher incidence in infants aged < 12 months. This might be due to the limited number of subjects we included, epidemiological changes due to the new emerging strains or differences in hospitalization criteria between centers. Although not statistically significant, the viral load of people infected by norovirus GII.2 was slightly higher than those infected with GII.4. This could also explain why outbreaks of norovirus GII.2 have more symptoms, and a shorter duration of symptoms.

Comparison of data from subjects infected with norovirus GII indicates that the age of symptomatic patients (median of 24 years) was significantly lower than that of asymptomatic patients (median of 46 years). This could be explained, as Parra et al*.* suggest, because adults have greater immunity to the various genotypes and that, although infected, in many cases do not present symptoms^30^. We also found that symptomatic patients had a higher viral load than asymptomatic ones, but we should not underestimate the viral load in asymptomatic patients, which was only slightly lower, meaning they may transmit the virus. Coinciding with the opinion of Shioda et al*.,* Ct values could be used to assess the disease burden^33^.

The disease burden and economic impact of noroviruses has motivated research to develop a norovirus vaccine. Vaccination could be of special importance to protect infants and young children, health care workers, food handlers, college students, military personnel, nursing home residents, and individuals dwelling in institutional settings^34^. However, specific studies to estimate the potential benefit of the vaccination in these collectives should be performed.

A limitation of this study is that, due to mildness of the disease or to problems in the detection and declaration of outbreaks, in some outbreaks the number of samples analyzed was low, so the statistical power to detect differences was also limited. Another limitation is the frequent mildness of the disease, which might have meant that some people affected did not consult their doctor, leading to an underestimation of cases.

In addition, because the surveys are carried out after the outbreak were detected and reported, the approach may involve recall bias and uncertainty in the associations' assumptions; however, epidemiological surveys are a commonly used tool which have been widely used in the investigation of norovirus outbreaks^35^. Another limitation was that it was not contemplated to carry out the genotyping of RdRp since the fragment sequenced for this work was sufficient to be able to specify the genotypes of the outbreaks.

## Conclusions

Our results show that norovirus outbreaks due to genotype GII.4 affect older populations. Norovirus outbreaks due to GII.2 and GII.4 are frequent in closed or semi-closed institutions, so exhaustive control and the implementation of adequate health measures by health staff in these institutions, and adequate hygiene in food handling is necessary. Symptomatic cases had a higher viral load, which suggests a greater capacity of transmission, although asymptomatic cases also presented relevant loads indicative of the capacity to transmit the virus.

## Data Availability

The datasets generated and analysed during the current study are available in the Mendeley Data repository, https://data.mendeley.com/datasets/nvpc7x7pvc/1. https://doi.org/10.17632/nvpc7x7pvc.1
